# FOXP3 inhibits cancer stem cell self-renewal via transcriptional repression of COX2 in colorectal cancer cells

**DOI:** 10.18632/oncotarget.17974

**Published:** 2017-05-18

**Authors:** Shuo Liu, Cun Zhang, Kuo Zhang, Yuan Gao, Zhaowei Wang, Xiaoju Li, Guang Cheng, Shuning Wang, Xiaochang Xue, Weina Li, Wei Zhang, Yingqi Zhang, Xianghui Xing, Meng Li, Qiang Hao

**Affiliations:** ^1^ The State Key Laboratory of Cancer Biology, Department of Biopharmaceutics, School of Pharmacy, The Fourth Military Medical University, Xi'an 710032, China; ^2^ Department of Pharmacogenomics, School of Pharmacy, The Fourth Military Medical University, Xi'an 710032, China; ^3^ State Key Laboratory of Military Stomatology, Department of Pediatric Dentistry, School of Stomatology, The Fourth Military Medical University, Xi'an 710032, China; ^4^ Department of Neurosurgery, Xijing Hospital, The Fourth Military Medical University, Xi'an 710032, China

**Keywords:** FOXP3, COX2, NFκB, cancer stem cell, transcriptional regulation

## Abstract

Colon cancer stem cell (cCSC) is considered as the seed cell of colon cancer initiation and metastasis. Cyclooxygenase-2 (COX2), a downstream target of NFκB, is found to be essential in promoting cancer stem cell renewal. However, how COX2 is dysregulated in cCSCs is largely unknown. In this study, we found that the expression of transcription factor FOXP3 was much lower in the spheroids than that in the parental tumor cells. Overexpression of FOXP3 significantly decreased the numbers of spheres, reduced the side population. Accordingly, FOXP3 expression decreased the tumor size and weight in the xenograft model. The tumor inhibitory effects of FOXP3 were rarely seen when COX2 was additionally knocked down. Mechanically, FOXP3 transcriptionally repressed COX2 expression via interacting with and thus inhibiting p65 activity on the putative NFκB response elements in COX2 promoter. Taken together, we here revealed possible involvement of FOXP3 in regulating cCSC self-renewal via tuning COX2 expression, and thus providing a new target for the eradication of colon cancer stem cells.

## INTRODUCTION

Colorectal cancer is one of the most frequent occurred cancers in the world. In the last several years, evidence has suggested that the capacity of initiating a tumor could be rather a unique characteristic of cells with stemness properties and the existence of colon cancer stem cells have been identified [1]. These so-called CSCs were originally identified through the expression of the CD133 glycoprotein using an antibody directed to its epitope AC133. Besides CD133, other cell surface markers, such as epithelial-specific antigen, CD44, CD166, Musashi-1, CD29, CD24, leucine-rich repeat-containing G-protein–coupled receptor 5, and aldehyde dehydrogenase 1, have been proposed. In addition to initiating and sustaining tumor growth, cCSCs are considered as the seed cell for chemoresistance and metastasis [2]. Therapeutically eradication of the cCSCs is believed to be the key to the treatment of colorectal cancer.

Colon cancer stem cells are maintained and affected by the niche, among which, the inflammatory microenvironment exert profound influence on the formation and evolution of cCSC. NFκB has been known as the most representative inflammatory factor and key transcription factor in the inflammatory microenvironment, which is involved in the physiological and pathological inflammation process. Previous studies have shown that NFκB could promote self-renewal of cCSC. However, the detailed mechanism how NFκB promotes the cancer stem cell self-renewal was largely unknown.

Cyclooxygenase-2 (COX2), also referred as prostaglandinendoperoxide synthase 2 (PTGS2), plays an essential role in promoting stem cell renewal and proliferation [3, 4]. It has been reported that COX2 was up-regulated in several kinds of CSCs [3, 5–7]. Given the well-known effects of COX-2 on stemness of cancer stem cells, which are now recognized as the key cell type for tumor metastasis and recurrence as described above, it is thus interesting to examine in molecular detail whether and how aberrant COX-2 expression regulates invasion and metastasis in the cCSC.

Abundant expression of FOXP3 have been identified in different cancers [8]. FOXP3 was also expressed by some non-lymphoid cells. Most of them identify FOXP3 as a tumor suppressor gene [9, 10]. We also report that FOXP3 interacts with NFκB, then inhibits expression of NFκB downstream target gene COX2, and exerts the influence on inhibiting tumor cells. All of these data suggest the existence of FOXP3-NFκB-COX2 axis and its implication in cCSC regulation.

In this study, we have found that FOXP3 is significantly down-regulated in cancer stem cell-like cancer cells. Forced expression of FOXP3 significantly decreased the self-renewal ability of cancer stem cells, as seen from the reduced number of colonospheres and the proportion of SP Cells, smaller successful rate of xenografts. Mechanistically, FOXP3 interacts with p65 and in turn inhibits NFκB mediated transcriptional activation of COX2. Our study here revealed a negative regulatory role of FOXP3 in tuning the self-renewal of cCSCs by inhibiting COX2 expression, which provides a new target and strategy for the removal of colon cancer stem cells.

## RESULTS

### Lower expression of FOXP3 in colon cancer stem cell

**Figure 1:**
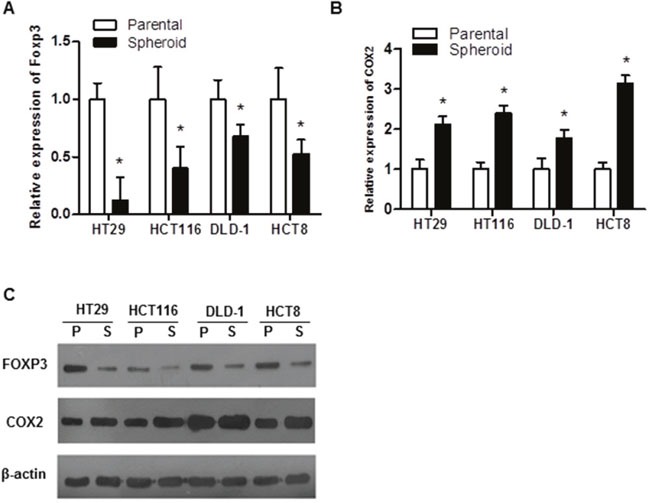
Expression of FOXP3 in colorectal cancer cell lines **(A, B)** FOXP3 **(A)** and COX2 **(B)** expression at mRNA level in the cell lines was detected by qRT-PCR, and β-actin served as an internal reference. All the experiments were done in triplicate and data were expressed as mean ±SD. * indicates p<0.05. **(C)** Expression of FOXP3 and COX2 at protein level was detected by Western blot, and β-actin served as a loading control. Data presented were representative of three different experiments.

In search of the molecular differences between parental cell and the colonospheres, we first focused on the expression of FOXP3, a molecule have been intensively studied by our lab [11–13]. Although increased FOXP3 activation has been revealed in many cancer types [8], its role in CSCs is largely unknown. To this end, we established the *in vitro* model of stem-like and sphere passage in different colon cancer cell lines. All the colorectal cancer cell lines produce sufficient colonospheres (Data not shown). Compared with the parental cells, the colonospheres displayed much lower expression of FOXP3 at both mRNA and protein levels (Figure 1A and 1C). Consistent with the reduced expression of FOXP3 in the colonospheres, much higher expression of COX2, a previously found downstream target negatively regulated by FOXP3 [12], was observed in the colonospheres (Figure 1B and 1C). All of these data indicate that FOXP3 and COX2 might involve in the regulation of the stemness of colon cancer stem cells.

### FOXP3 suppresses self-renewal in colon cancer stem cell

**Figure 2:**
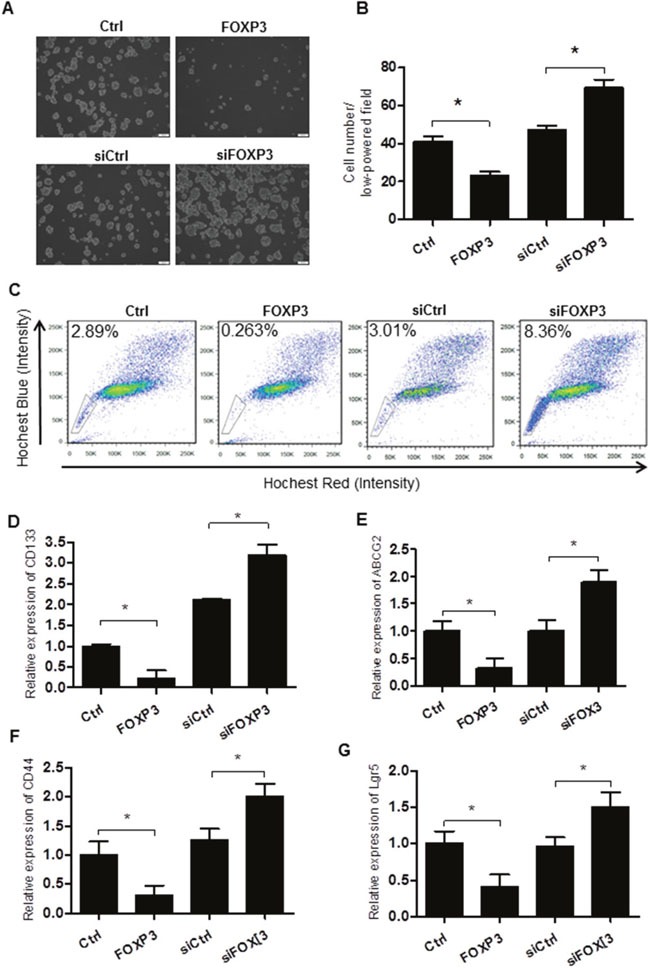
FOXP3 inhibits the self-renewal of the colorectal cancer stem cells **(A)** Cells were infected with FOXP3 overexpressing or knockdown virus and corresponding controls as indicated. Decreased colonosphere formation in FOXP3 overexpressing cells compared with the control cells, while increased colonosphere formation in FOXP3 knockdown cells compared with the control cells. Bar = 100 μm. **(B)** Quantification data of Figure 2A. **(C)** Flow cytometry analysis of the side population in cells treated same as above. Data presented were representative of three different experiments. **(D-G)** qPCR analysis of the stem cell marker CD133 **(D)**, ABCG2 **(E)**, CD44 **(F)** and Lgr5 **(G)** in cells treated same as above. β-actin served as an internal reference. All the experiments were done in triplicate and data were expressed as mean ±SD. * indicates p<0.05.

In view of the above data, we hypothesized that FOXP3 could suppress self-renewal ability of colon cancer stem cell. Side population analysis by flow cytometry was included, and verapamil treatment confirmed the gated cells were indeed the side population (Supplementary Figure 1). Next, we infected colon cancer cell HT29 with FOXP3 overexpression or interference viruses. As expected, forced expression of FOXP3 was observed at both mRNA level and protein levels significantly (Supplementary Figure 2). Consistently, FOXP3 overexpression significantly decreased the number of colonosphere formation (Figure 2A and 2B) and the SP percentage (Figure 2C). Meanwhile, qPCR analysis of the putative stem cell markers revealed that CD133, Lgr5, CD44, and ABCG2 expression decreased at mRNA level upon FOXP3 expression (Figure 2D-2G). In contrast, knockdown of FOXP3 significantly increased the formation of colonospheres, side population proportion, together with the increased marker gene expression (Figure 2).

**Figure 3:**
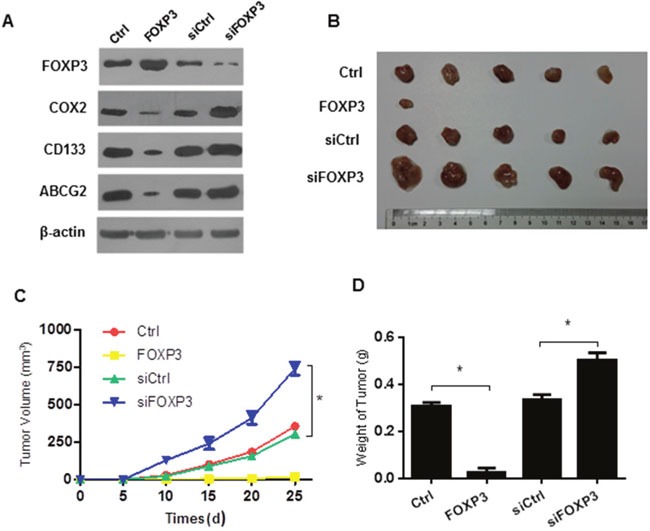
FOXP3 inhibits tumor formation in the xenograft model **(A)** Expression of COX2, FOXP3,ABCG2 and CD133 in the different transplanted cells were was detected by Western blot, and β-actin served as a loading control. Data presented were representative of three different experiments. **(B)** Cells with indicated treatments were inoculated in the nude mice and tumor volumes of the above xenografts were monitored every five days. Data were expressed as mean ±SD. * indicates p<0.05. **(C)** Cells with indicated treatments were inoculated in the nude mice and tumors were excised 4 weeks later. The sizes of the tumors were compared among groups. n=5 per group. **(D)** Tumor weight of the above tumor xenografts. Data were expressed as mean ±SD. * indicates p<0.05.

Expression of COX2, cancer stem cell marker CD133 and drug resistance gene ABCG2 in the above cells, were further confirmed by Western blot before *in vivo* xenograft analysis (Figure 3A). Tumor xenograft model further confirmed that about 50,000 HT29 cells could form tumors in nude mice, while the same number of HT29 cells with FOXP3 transfection could not form detectable tumors (Figure 3B-3D). In contrast, knockdown of FOXP3 significantly increased the tumor formation and volume (Figure 3A-3D). All of these data confirmed the negative regulatory role of FOXP3 on the self-renewal ability of colon cancer stem cells.

### FOXP3 decreases COX2 expression by interaction with NFκB on COX2 promoter

**Figure 4:**
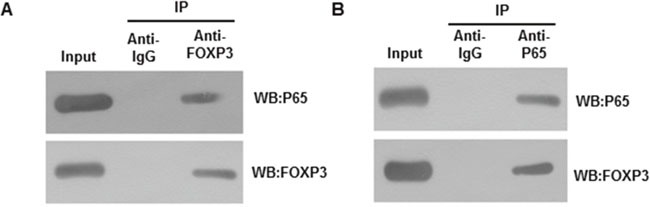
Interaction between FOXP3 and p65 **(A)** Cell lysis was immuneprecipitated with anti-FOXP3 antibody and the immunoprecipitated complex were analyzed by indicated antibodies. Data presented were representative of three different experiments. **(B)** Cell lysis was immuneprecipitated with anti-p65 antibody and the immunoprecipitated complex were analyzed by indicated antibodies. Data presented were representative of three different experiments.

**Figure 5:**
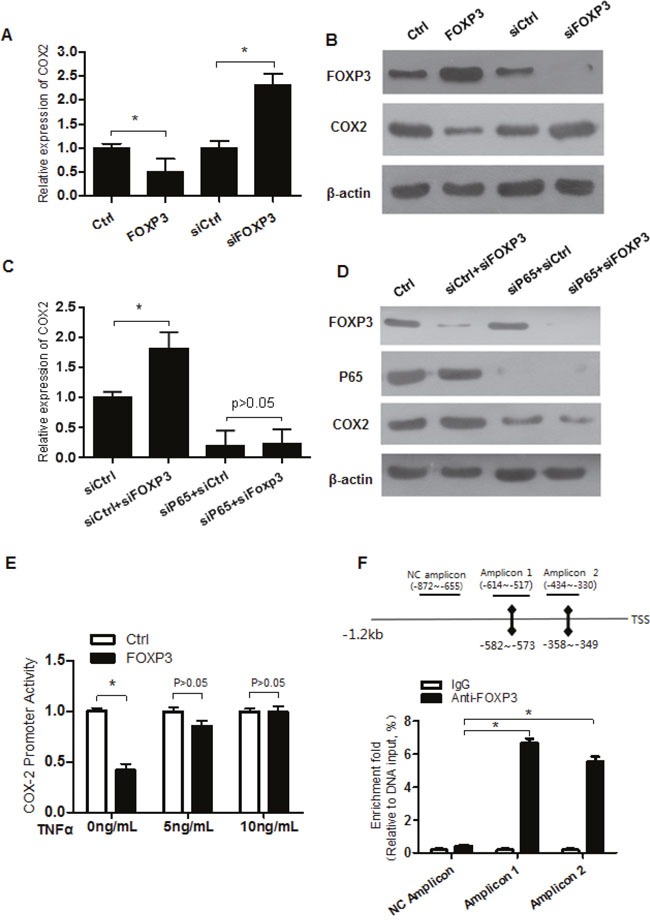
FOXP3 represses COX2 expression via p65 **(A)** COX2 expression at mRNA level in the cell lines with FOXP3 overexpression or knockdown was detected by qRT-PCR, and β-actin served as an internal reference. All the experiments were done in triplicate and data were expressed as mean ±SD. * indicates p<0.05. **(B)** Expression of FOXP3 and COX2 at protein level in the cells with FOXP3 overexpression or knockdown was detected by Western blot, and β-actin served as a loading control. Data presented were representative of three different experiments. **(C)** Cells with FOXP3 knockdown were additionally treated with si-p65 or the control. COX2 expression at mRNA level was analyzed by qPCR. β-actin served as an internal reference. All the experiments were done in triplicate and data were expressed as mean ±SD. * indicates p<0.05. **(D)** Cells were treated same as above. Expression of FOXP3, p65 and COX2 at protein level was detected by Western blot, and β-actin served as a loading control. Data presented were representative of three different experiments. **(E)** FOXP3 inhibits COX2 promoter activity in a p65 dependent manner. COX2 promoter reporter vector were co-transfected with FOXP3 or control vector, together with the internal control pRL-TK. Additionally, cells were treated with different doses of TNFα. Relative luciferase activity was analyzed. All the experiments were done in triplicate and data were expressed as mean ±SD. * indicates p<0.05. **(F)** ChIP analysis of FOXP3 interaction with COX2 promoter on the NFκB response elements. The putative NFκB response elements were indicated. A nearby region without NFκB binding sites was also included as a non-specific binding control. All the experiments were done in triplicate and data were expressed as mean ±SD. * indicates p<0.05.

We have reported that FOXP3 inhibited NFκB activity [12]. Similar as the positive control IL6, TNFα induced similar upregulation of COX2 and enrichment of p65 on the promoter (Supplementary Figure 3), confirming that COX2 is a real target gene of NFκB. Consistent with previous findings, we continued to found that the remaining FOXP3 still interacted with p65 in sphere cells (Figure 4A and 4B). Moreover, transfection of FOXP3 repressed COX2 expression at both mRNA and protein levels. In contrast, knockdown of endogenous FOXP3 increased COX2 expression at both mRNA and protein levels (Figure 5A and 5B). To further confirm whether FOXP3 transcriptionally represses COX2 expression via p65, p65 was simultaneously knocked down. Upon p65 knockdown, the promoting effects on COX2 expression by FOXP3 knockdown couldn't be seen anymore, though inhibiting p65 alone decreased expression of COX2 (Figure 5C and 5D). Consistent with the endogenous expression, luciferase reporter activity assay revealed that FOXP3 inhibited the promoter of COX2 activity, which can be blocked by co-expression of p65 (Figure 5E). The translocation of p65 upon TNFα was confirmed by Western blot (Supplementary Figure 4). ChIP assay revealed occupation of FOXP3 on the NFκB binding sites of the COX2 promoter in an NFκB dependent binding manner (Figure 5F). Truncated deletion and potential NFκB-binding site mutation of COX2 promoter revealed that both the NFκB-binding sites are required for p65 interaction and thus optimal repression by FOXP3 (Supplementary Figure 5A and 5B). All of these data implied that expression of COX2 was regulated by FOXP3-NFκB interaction on the COX2 promoter.

### FOXP3 inhibits self-renewal by repressing COX2

**Figure 6:**
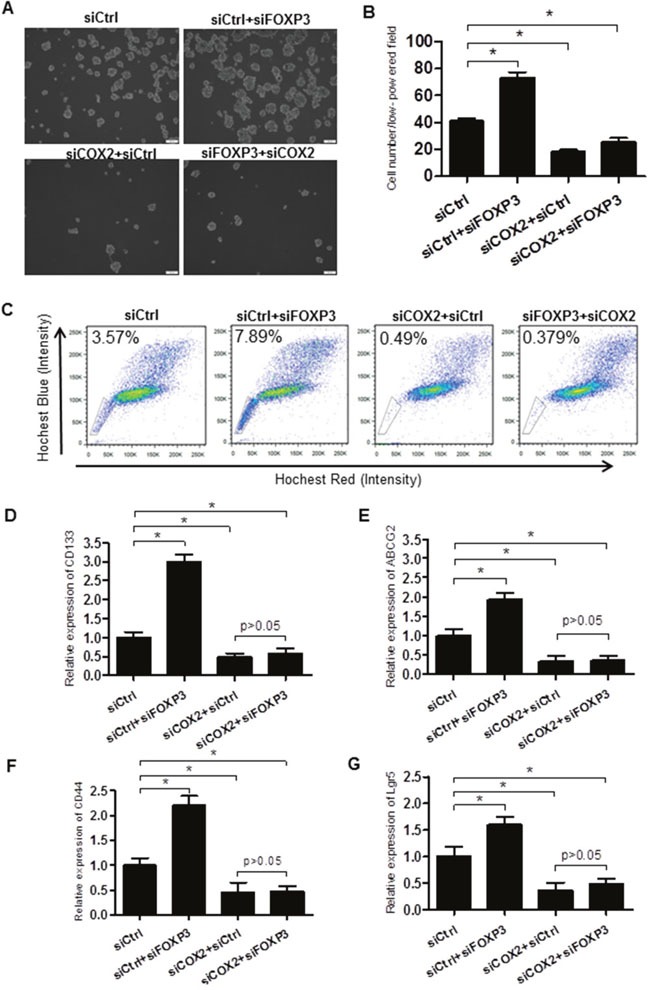
FOXP3 inhibits the self-renewal of the colorectal cancer stem cells via COX2 repression **(A)** Cells were treated as indicated. Increased colonosphere formation were observed in FOXP3 knockdown cells compared with the control cells, which was totally blocked when COX2 was additionally knocked down. Bar = 100 μm. **(B)** Quantification data of Figure 6A. **(C)** Flow cytometry analysis of the side population in cells treated same as above. Data presented were representative of three different experiments. **(D-G)** qPCR analysis of the stem cell marker CD133 **(D)**, ABCG2 **(E)**, CD44 **(F)** and Lgr5 **(G)** in cells treated same as above. β-actin served as an internal reference. All the experiments were done in triplicate and data were expressed as mean ±SD. * indicates p<0.05.

**Figure 7:**
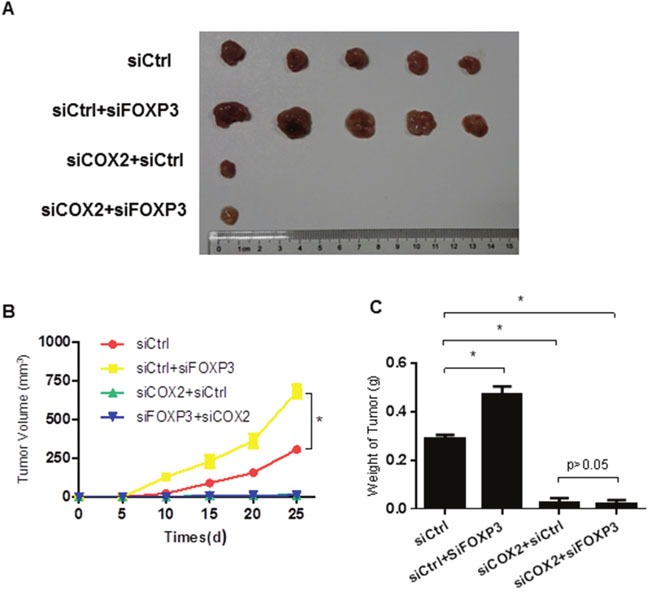
FOXP3 inhibits tumor formation via COX2 in the xenograft model **(A)** Cells with indicated treatments were inoculated in the nude mice and tumor volumes of the above xenografts were monitored every five days. Data were expressed as mean ±SD. * indicates p<0.05. **(B)** Cells with indicated treatments were inoculated in the nude mice and tumors were excised 4 weeks later. The sizes of the tumors were compared among groups. n=5 per group. **(C)** Tumor weight of the above tumor xenografts. Data were expressed as mean ±SD. * indicates p<0.05.

All of the above findings suggest that FOXP3 inhibited the expression of COX2 by inhibiting NFκB activity. We next asked whether COX2 might be the key factor mediating the effects of FOXP3 on cCSC self-renewal. As expected, inhibition of COX2 alone significantly decreased the colonosphere formation (Figure 6A and 6B), the proportion of SP Cells (Figure 6C) and marker gene expression (Figure 6D-6G), together with reduced tumor volume (Figure 7). However, no combinatory effects on colonosphere, proportion of SP Cells, marker gene expression and tumor formation were observed when cells were treated with both FOXP3 transfection and COX2 inhibition (Figure 6 and Figure 7). All of these above results suggest that FOXP3 inhibits colon cancer stem cell self-renewal in a COX2 dependent manner.

## DISCUSSION

Colorectal cancer is the second leading cause of cancer-related death in the world [14]. Since cCSC is considered as the seed cell for cancer growth, metastasis and chemoresistance, improving the understanding of cCSC behavior and self-renewal mechanism could lead to novel therapies. In this study, we have found that transcriptional factor FOXP3 is intrinsically down-regulated in stem cell-like cancer cells. Forced expression of FOXP3 significantly decreased the self-renewal ability of cancer stem cells, and thus the tumorigenic capacity. Mechanistically, FOXP3 interacts with p65 and in turn inhibits NF-κB mediated transcriptional activation of COX2.

Niches are the physical environments that maintain SCs in a variety of tissues, including human colon [15]. In the context of colorectal, inflammatory cells and the cytokines secreted are key characteristics of the cCSC niche. As a dominant player in the inflammation, COX2 has been found to play an important role in angiogenesis and metastasis in the context of colorectal cancer. We here further identified that COX2 expression is one of the essential factors responsible for the stemness of colorectal cancer cells. Since stemness is the origin of metastasis and angiogenesis, it is thus reasonable to conclude that COX2 promotes metastasis and angiogenesis at least partially via promoting stemness [16]. Previous studies have demonstrate that Wnt, TGFβ and Notch pathways contribute to colorectal cancer stemness and colonosphere formation [17–19]. It is thus reasonable to speculate that COX2 could integrate Wnt, TGFβ and Notch pathways. Further characterization of the role of COX2 in the stemness controlling network will better our understanding of the oncogenic mechanisms of COX2.

COX2 itself is fine-tuned by both negative and positive mechanisms. Previous studies have found that NFκB is one of the most well-studied transcription factors that induce COX2 expression [20]. Here we found that FOXP3 interacts with the p65 subunit of NFκB on the promoter of COX2, subsequently forming an inhibitory complex. And thus reduction of FOXP3 results in augmented NFκB activity and thus aggravated expression of COX2. As to how FOXP3 negatively regulates NFκB, there are multiple explanations. FOXP3 was found to be able to alter histone modifications around its binding sites [21], and thus interaction between FOXP3 and NFκB might lead to the histone modification alteration in the COX2 locus. It is thus highly possible that the interaction between FOXP3 and NFκB on the COX2 promoter might recruit negative epigenetic regulators, thus turning down gene expression. Alternatively, interaction between FOXP3 and NFκB on the COX2 promoter might induce a repressive allosteric conformation of NFκB. It is important to note that besides negative regulation of COX2, there might be also other targets mediating the tumor suppressor function of FOXP3-NFκB interaction. In fact, there are many other target genes of NFκB, such as IL-6 and iNOS, all of these genes are also important in the cancer stem cell self-renewal, chemoresistance and/or metastasis of tumors [22–24]. In addition, we have shown that the interaction between FOXP3 and NFκB can also drive the FOXP3 away from the promoter of its target gene p21 [13]. Future studies unraveling the detailed downstream effects of FOXP3-NFκB interaction on cancer stem cells would certainly broaden our understanding on how cancer stem cells are maintained and evolved.

Taken together, we have found that FOXP3 is significantly down-regulated in cancer stem cell-like cancer cells. Forced expression of FOXP3 significantly decreased the self-renewal ability of cancer stem cells. Mechanistically, FOXP3 interacts with p65 and in turn inhibits NF-κB mediated transcriptional activation of COX2. Our study here revealed a negative regulatory role of FOXP3 in tuning the self-renewal of cCSCs by inhibiting COX2 expression, which provides a new target and strategy for the removal of colon cancer stem cells. We expect the development of activators targeting FOXP3 for colorectal cancer treatment.

## MATERIALS AND METHODS

### Cell culture and spheroid body formation

The human colorectal cancer cell line HT29 was purchased from Cell Bank of Type Culture Collection of the Chinese Academy of Sciences (Shanghai Institute of Cell Biology, Chinese Academy of Sciences). The human colorectal cancer cell line HCT116, DLD1 and HCT8 were granted by Department of Immunology of the Fourth Military Medical University. HT29 and HCT116 cell lines were maintained in McCOY's 5A medium (MACGENE)supplemented with 10% fetal calf serum (Gibco), DLD1 and HCT8 cell lines were maintained in RPMI 1640 medium (HyClone) supplemented with 10% fetal calf serum(Gibco). These cell lines were incubated in a humidified atmosphere with 5% CO_2_ at 37°C. To generate spheroid bodies, single cells were seeded on ultralow attachment plates (Corning) at a density of 1,000 cells/mL. Cells were maintained in DMEM/F12 (Gibco) supplemented with B-27 (Gibco), basic fibroblast growth factor (PeproTech), epidermal growth factor (PeproTech), 0.4% bovine serum albumin (Sigma), and 4 mg/mL Insulin (Sigma) and were incubated in a humidified atmosphere with 5% CO_2_ at 37°C. The average number of spheroids (with a size>50μm) was calculated 5-7 days after seeding under microscope.

### Lentivirus infection

To overexpress and interfere with FOXP3, COX2 and p65, overexpression and shRNA viruses were constructed and packaged using pWPI or pLKO.1 system. The human colorectal cancer cell line HT29 was planted in 24-well plants, 5×10^4^cells and 0.5 milliliter complete medium per well. The cells were incubated in a humidified atmosphere with 5% CO_2_ at 37°C for 12 hours. The complete medium was aspirated and different virus diluents were added to the 24-well plants, 12 hours after infection, the virus diluent was aspirated and 0.5 milliliter complete medium was added. The cells were incubated for 24-48 hours, at the end of incubation, the cells were harvested and digested into single cells for spheroid body formation.

### Quantitative real-time polymerase chain reaction

Total RNA of the cells with different treatment was extracted by using Trizol(Takara). cDNA was synthesized by reverse transcription using PrimeScript™ RT Master Mix(Takara), according to the manufacturers’ guidelines. The Quantitative real-time polymerase chain reaction was performed by using an AB 7500 system and SYBR Green PCR kit (Takara). The relative expression of mRNA of different targets were normalized to GAPDH mRNA and analyzed with the control using the 2 ^−ΔΔCt^. All experiments were performed in triplicate. The primer sequences are listed in Supplementary Table 1.

### Western blotting analysis

Cells with different treatment were prepared by removing the media from the cells and washing with cold phosphate-buffered saline (PBS). Cells were lysed with RIPA buffer containing proteinase inhibitor on ice for 30 min. The mixture was centrifuged at 12,000 rpm for 10 min at 4°C, the supernatant (total protein) was obtained and were quantified using BCA assay. The mixture of total protein and loading buffer was boiled for 5 min and centrifuged at 12,000 rpm for 5 min at room temperature. Samples with equal amounts of protein were separated on sodium dodecyl sulfate-polyacrylamide gels and then transferred into PVDF membranes. To reduce nonspecific binding, the PVDF membranes were then incubated in 5% non-fat milk in Tris-buffered saline with 0.1% Tween-20. The membranes were incubated with the following antibodies against FOXP3, P65, COX-2, ABCG2, Lamin B and β-actin overnight at 4 °C at a dilution as recommended by the manufacturer. Subsequently, the membranes were then washed in TBST and were incubated with secondary antibodies for 50 mim at room temperature at a dilution of 1:5000. Finally, the relative protein abundance was visualized by using ECL.

### Side population

Side population cells were determined by flow cytometry as described before [25]. Briefly, all the cells were digested into single cell suspension, clone cells with Trypsin-EDTA (HyClone) and Spheres with Accutase (Sigma). The single-cell suspension (10^6^ cells/ml) were incubated with Hoechst 33342 (5 μg/ml) at 37 °C for 60 min with or without the ABCG2 transporter inhibitor Verapamil (100 μM). During the incubation, the cells were shaken every 5 min. After incubating, the cells were washed by cold PBS and then suspended in Hanks’ balanced saline solution (HBSS; HyClone) containing 2% FBS and 1 mM HEPES and kept in dark at 4°C. Propridium iodide (2μg/mL) was added to exclude dead cells during flow cytometry. After filtered with a 400-mesh screen filter to ensure a single cell suspension, cells were analyzed using the fluorescence-activated cell sorting (FACS)-Vantage SE (BD Biosciences).

### Xenografts

All the animal experimental protocols in this study were monitored and approved by the Institutional Animal Care and Use Committee of the Fourth Military Medical University. The least number of animals necessary for getting reliable results was used, and most attention was paid to animal rights and health protection. Briefly, 4-week-old female Balb/c nude mice (Shanghai, China) were purchased from the Forth Military Medical University Experimental Center and were randomly assigned to different groups. After one week of acclimatization, 1000 cells were suspended in 200 μL of normal saline containing 25% Matrigel (Corning) and injected subcutaneously into nude mice. The length and width of tumors were measured every 3-5 days with calipers. Tumor volume was estimated based on the formula: Volume= (Length×Width^2^)/2. After four weeks, tumors were harvested and the tumor volume and tumor weight were recorded. The tumor volume and weight was expressed as mean±SD of all the mice injected in each group.

### Co-immunoprecipitation (Co-IP)

IP antibody P65(Cell Signaling) or FOXP3(Abcam) 10μL diluted in 200μL PBS with Tween20 was added to 50μL Dynabeads Protein G(Life technologies) and was incubated with rotation overnight at 4°C. Then the supernatant was removed, the Dynabeads-antibody complex was washed by PBS with Tween20. The Dynabeads-antibody complex was resuspended in the protein sample containing the antigen and was incubated with rotation for 2 hours at 4°C. After that, the supernatant was removed, the complex was resuspended in 2×Loading buffer and boiled then centrifuged at 12,000g for 3min. Then the western blot was performed.

### Luciferase assay

HT29 cells were transfected with either control vector or FOXP3 using Lipofectamine 2000 together with indicated COX2 promoter luciferase reporter vectors and Renilla-luc control vector. Twenty-four hours after transfection, cells were digested into single cells and were cultured to generate spheres. Additionally, cells were treated with different doses of TNFα. 3 days after transfection, the spheres were lysed using passive lysis buffer, and the luciferase activity was measured using Dual Luciferase kit (Promega) according to manufacturer's instructions.

### Chromatin immunoprecipitation (ChIP)

Cells were fixed with 1% formaldehyde and then were sonicated to produce 200–1000 bp DNA fragments. ChIP was carried out according to the manufacturer's guidelines (Millipore). Anti-FOXP3 antibody and control human IgG were used. The amounts of the specific DNA fragment was then quantified by real-time polymerase chain reaction and normalized against the input. The primers are listed in Supplementary Table 2.

### Statistics

Statistical analyses were performed using SPSS 16.0 and GraphPad Prism 5.0. The data were expressed as means ± SD. Statistical significance was analyzed by non-paired t-test and expressed as p value. A p-value less than 0.05 was considered significant.

## SUPPLEMENTARY MATERIALS FIGURES AND TABLES


