# Supplementary material for: FOXP3 inhibits cancer stem cell self-renewal via transcriptional repression of COX2 in colorectal cancer cells
Source: Oncotarget. 2017 May 18;8(27):44694–704. doi: 10.18632/oncotarget.17974 (PMC5546511; doi:10.18632/oncotarget.17974)
Supplement: Supplementary file 1 [file oncotarget-08-44694-s001.pdf]

FOXP3 inhibits cancer stem cell self-renewal via transcriptional repression of COX2 in colorectal cancer cells

SUPPLEMENTARY MATERIALS

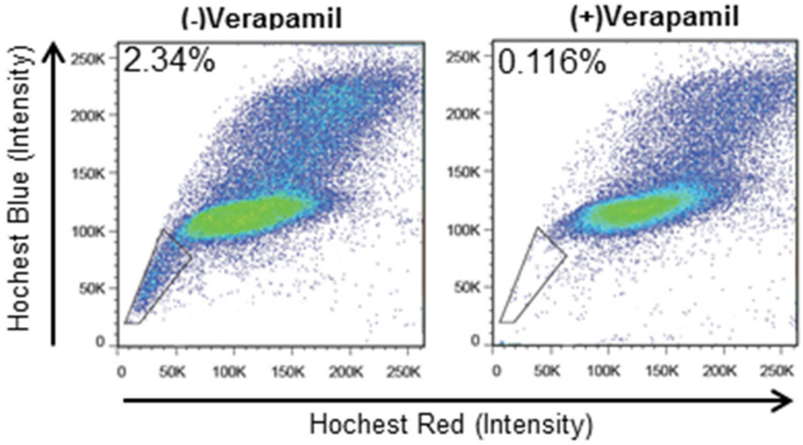

Supplementary Figure 1: Verapamil inhibits the percentage of the side population (SP) cells. Cells were labeled with the Hoechst 33342 in the absence (left panel) or presence of 50μM verapamil (right panel), followed by SP analysis. These experiments were repeated at least three times with similar results.

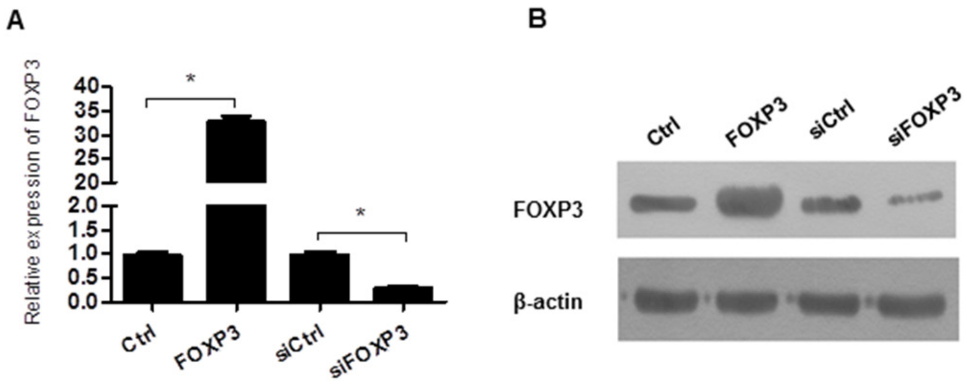

Supplementary Figure 2: Overexpression and knockdown efficiency of FOXP3. **(A, B)** FOXP3 expression at mRNA level **(A)** and protein level **(B)** in the cells treated as indicated was detected by qRT-PCR and Western Blot respectively, and GAPDH or β-actin served as an internal reference. All the experiments were done in triplicate and data were expressed as mean ±SD. * indicates $p<0.05$.

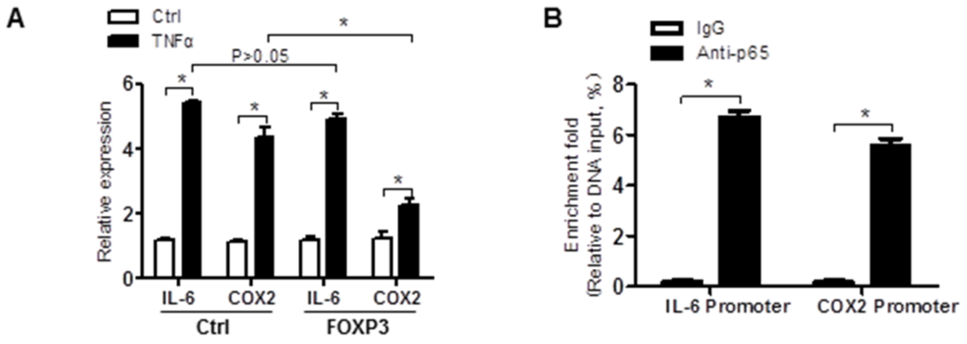

Supplementary Figure 3: FOXP3 represses COX2 expression via p65. **(A)** Sphere cells infected with FOXP3 overexpression virus or corresponding control were treated with or without TNFα. IL-6 and COX2 expression at mRNA level were detected by qRT-PCR, and GAPDH served as an internal reference. All the experiments were done in triplicate and data were expressed as mean ±SD. * indicates $p<0.05$. **(B)** ChIP analysis of p65 interaction with IL-6 promoter and COX2 promoter on the NFκB response elements. All the experiments were done in triplicate and data were expressed as mean ±SD. * indicates $p<0.05$.

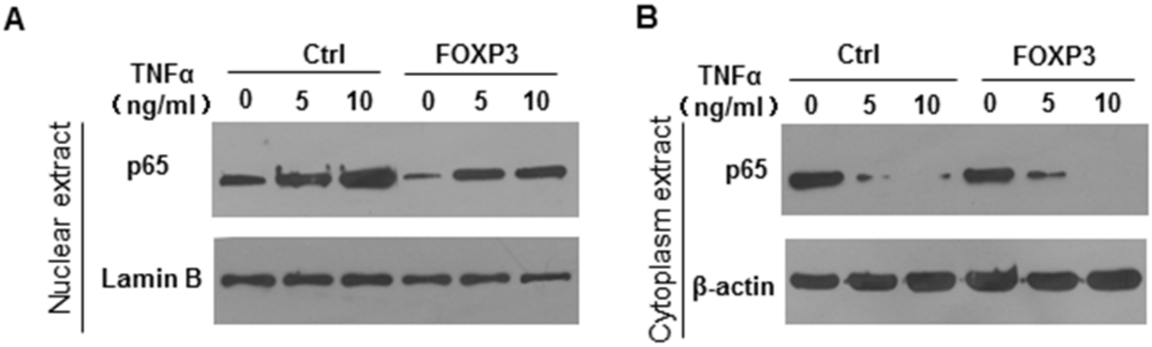

Supplementary Figure 4: TNFα induces nuclear translocation of p65. Sphere cells infected with FOXP3 overexpression virus or corresponding control were treated with different dose of TNFα. Western blot analysis of the p65 expression in nuclear extract **(A)** and cytoplasm extract **(B)** of cells. Lamin B and β-actin served as internal references.

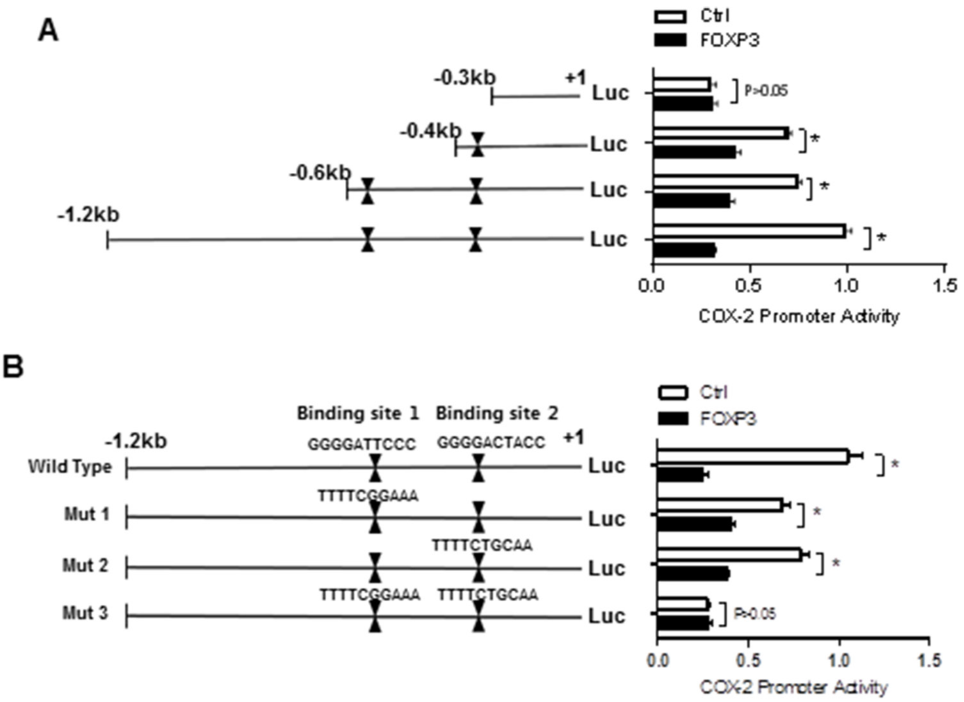

Supplementary Figure 5: Transcriptional repression of COX-2 expression by FOXP3. **(A)** Deletion of the NFκB-binding sites in COX-2 promoter region prevented FOXP3-mediated suppression. Luciferase activity was measured in sphere cells. All the experiments were done in triplicate and data were expressed as mean ±SD. * indicates $p<0.05$. **(B)** Site-directed mutagenesis of NFκB-binding sites in the COX-2 promoter region prevented FOXP3-mediated suppression. Luciferase activity was measured in sphere cells. All the experiments were done in triplicate and data were expressed as mean ±SD. * indicates $p<0.05$.

Supplementary Table 1: Primers used in RT-PCR

Primer name	Forward	Reverse
GAPDH	GACCTGACCTGCCGTCTA	AGGAGTGGGTGTCGCTGT
FOXP3	TTCGAAGAGCCAGAGGACTT	ATGGCACTCAGCTTCTCCTT
COX2	CTGGCGCTCAGCCATACAG	CGCACTTATACTGGTCAAATCCC
CD133	CACTTACGGCACTCTTCACCTG	CAATTCACTGCTTATGCTTCCA
CD44	AAAGGAGCAGCACTTCAGGA	TGTGTCTTGGTCTCTGGTAGC
Lgr5	CTCCCAGGTCTGGTGTGTTG	GAGGTCTAGGTAGGAGGTGAAG
ABCG2	CAGGTGGAGGCAAATCTTCGT	ACCCTGTTAATCCGTTCGTTTT
IL-6	ACTCACCTCTTCAGAACGAATTG	CCATCTTTGGAAGGTTCAGGTTG
β-actin	CTCCATCCTGGCCTCGCTGT	GCTGTCACCTTCACCGTTCC

Supplementary Table 2: Primers used in ChIP

Primer name	Sense	antisense
NC amplicon	GGTGAAGGTACGGAGAACAG	GGTTAAGAAAGGCTGACATG
Amplicon 1	TACAGACCAGACACGGCGG	AAGGACACTTGGCTTCCTCTCCA
Amplicon 2	AAAGACATCTGGCGGAAACCTGTG	CCAATTTGGGAGCAGAGGG
IL6	AGCACTGGCAGCACAAGGCAAAC	CAAGCCTGGGATTATGAAGAAGG
